# Early Postnatal *Shank3* Downregulation in the Nucleus Accumbens Impairs Performance in Social Conditioning Paradigms in Male Mice

**DOI:** 10.1111/ejn.70203

**Published:** 2025-08-04

**Authors:** Alessandro Contestabile, Giulia Casarotto, Benoit Girard, Beatrice Righetti, Clément Solié, Camilla Bellone, Stamatina Tzanoulinou

**Affiliations:** ^1^ Department of Basic Neuroscience University of Geneva Geneva Switzerland; ^2^ Department of Neuroscience Karolinska Institutet, Biomedicum Stockholm Sweden; ^3^ Department of Neurosurgery, Klinikum rechts der Isar Technical University of Munich Munich Germany; ^4^ Brain Plasticity Unit, CNRS, ESPCI Paris PSL Research University Paris France; ^5^ Department of Biomedical Sciences, Faculty of Biology and Medicine University of Lausanne Lausanne Switzerland

**Keywords:** autism spectrum disorder, behavioral classification, nucleus accumbens, Shank3, social conditioned place preference, social instrumental task

## Abstract

Autism spectrum disorder (ASD) is a heterogeneous neurodevelopmental disorder characterized by reduced social interactions, as well as repetitive behaviors and restricted interests. Mutations in *SHANK3*, a scaffolding protein located postsynaptically at excitatory synapses, are associated with ASD, schizophrenia, and intellectual disability in humans. Similar autism‐like phenotypes have been observed in *Shank3*‐deficient rodent models. The mesolimbic dopamine pathway appears to be particularly sensitive to *Shank3* disruptions. We have previously shown that *Shank3* downregulation in the nucleus accumbens (NAc) (*Shank3‐NAcKD*) during early postnatal development impaired social preference in the three‐chamber test. Here, we aimed to assess whether this *Shank3* downregulation would lead to deficits in social conditioning paradigms. Specifically, using the social instrumental task (SIT), we found that *Shank3‐NAcKD* male mice performed fewer lever presses to gain access to social interaction with a nonfamiliar juvenile mouse. Moreover, these mice failed to develop a preference for the chamber associated with social stimuli in a conditioned place preference (CPP) task. Unsupervised analysis of locomotor motifs during CPP revealed distinct exploratory strategies, with an altered allocation of exploratory behaviors between the socially paired and unpaired chambers, suggesting a suboptimal direction of exploration towards relevant social‐associated cues. Our current data expand on our previous research to understand the involvement of mesolimbic *Shank3* expression in autism‐like phenotypes. Additionally, our results underline that local *Shank3* manipulation during early postnatal life leads to intricate social behavior deficits, highlighting the need for an in‐depth dissection of behavioral phenotypes in rodent models of ASD.

## Graphical abstract

Early postnatal *Shank3* downregulation in male mice leads to deficits in a social instrumental task (SIT), with mice performing fewer lever presses to gain access to social interaction with a nonfamiliar younger mouse. These mice also fail to develop a preference for the chamber associated with social stimuli in a conditioned place preference (CPP) task. Our results highlight the need for an in‐depth dissection of behavioral phenotypes in rodent models of autism.

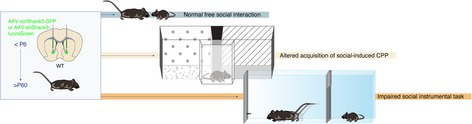

**Abbreviations**
ASD: autism spectrum disorder
CPP: conditioned place preference
D1R‐MSNs: D1 receptor‐expressing medium spiny neurons
DA: dopamine
NAc: nucleus accumbens
*Shank3‐NAcKD*
: Shank3 nucleus accumbens knockdown
SIT: social instrumental task
VTA: ventral tegmental area

## 1 Introduction

Autism spectrum disorder (ASD) is a heterogeneous and heritable neurodevelopmental disorder, characterized by perturbed social interactions, repetitive behaviors, and restricted interests, with an estimated prevalence of 1 in 132 (Baxter et al. 2015; DSM 2013). Among the genes implicated in ASD, *SHANK3* has received significant attention due to its strong association with autism, schizophrenia, and intellectual disability (Phelan and McDermid 2012; Zoghbi and Bear 2012; Bariselli and Bellone 2017). Mutations in *Shank3* have been extensively validated in rodent models, where they are linked to autism‐like phenotypes, including impaired social interactions (Peca et al. 2011; Mei et al. 2016).

*SHANK3* encodes a scaffolding protein localized at the excitatory postsynaptic compartments, where it coordinates interactions with glutamate receptors and other postsynaptic proteins, thus playing a pivotal role in synaptic transmission and plasticity (Jiang and Ehlers 2013; Delling and Boeckers 2021). Animal studies using *Shank3* knockout models have demonstrated that disruptions in *Shank3* expression result in profound impairments in social behaviors and can lead to cognitive and motor deficits (Peca et al. 2011; Kouser et al. 2013). However, whether *Shank3* downregulation contributes to more specific deficits in social conditioning paradigms—pavlovian and operant—remains less explored. Research from animal models can offer potent insights to bridge this significant gap in our knowledge, especially as social motivation deficits have been hypothesized to contribute further to suboptimal learning from social contexts in ASD patients (Vivanti and Nuske 2017; Bushwick 2001).

The mesolimbic dopamine pathway seems to be particularly sensitive to *Shank3* disruptions, as we and others have shown (Bariselli et al. 2016; Mei et al. 2016; Wang et al. 2017; Tzanoulinou et al. 2022). Previous studies have shown that Shank3 expression in rat and mouse brains increases gradually early in development, with the peak of expression within the first 2 to 4 weeks, followed by a decrease during adulthood (Wang et al. 2014; Bockers et al. 2001). Downregulating *Shank3* during the first postnatal week in the ventral tegmental area (VTA) has been shown to lead to cell‐specific changes in excitatory transmission in this brain area (Bariselli et al. 2016). Moreover, nucleus accumbens (NAc)‐specific *Shank3* downregulation during the same developmental period was reported to lead to hyperexcitability in D1 receptor‐expressing medium spiny neurons (D1R‐MSNs) (Tzanoulinou et al. 2022).

This local downregulation of *Shank3* in the NAc during early postnatal life leads to impaired sociability as denoted by a reduction in social preference during the three‐chamber test (Tzanoulinou et al. 2022). Interestingly, this reduced social preference was driven not only by decreased exploration of an unfamiliar juvenile mouse but also by increased exploration of the inanimate object. Orientation and interest in nonsocial stimuli in children have been shown to predict ASD diagnoses (Vivanti and Nuske 2017; Pierce et al. 2011). Thus, whereas previous findings suggest decreased sociability following early NAc *Shank3* downregulation, it remains unclear whether this also impairs social interaction or social conditioning paradigms, notably when no other “competing” stimulus is included in the experimental design, that is, where no choice is required between social and inanimate targets.

To address these questions, we first assessed performance in a simple free interaction paradigm following early postnatal NAc *Shank3* downregulation (*Shank3‐NAcKD*). We did not observe differences in the exploration of another sex‐matched juvenile conspecific between control and *Shank3‐NAcKD* mice. We next examined performance in social conditioning paradigms in these mice. Our results indicate that early *Shank3‐NAcKD* leads to impairments in both instrumental and associative conditioning paradigms. Collectively, our current study expands and enriches our previous work and contributes to refining our understanding of the role of *Shank3* in social conditioning in a brain region‐specific manner.

## 2 Materials and Methods

### 2.1 Animals

Pregnant female C57BL/6J mice were purchased from Charles River Laboratories France and housed in the institutional animal facility under standard 12 h/12 h light/dark cycles with food and water ad libitum. Male pups were stereotactically injected at P5 or P6 as described below and returned immediately to the dam and litter until weaning at P21. Experimental animals were group‐housed (two to five per cage) and were behaviorally tested after P60 as stated in the corresponding sections in the following paragraphs. All animals were prepared according to the method previously described and validated in Tzanoulinou et al. (2022)—see more details below. An adequate number of litters was used for each cohort (see Table S1 for cohort details), and mice were randomly assigned to either the scr‐ or sh*Shank3* group to minimize potential litter effects. A subset of the cohorts included in this study has also been used in previously published work involving different behavioral tests (Tzanoulinou et al. 2022). Detailed information regarding the behavioral assays conducted in both the current and previous study, as well as the number of litters used, can be found in Table S1. Younger nonfamiliar male mice (3–5 weeks; sex‐matched) were used as stimuli animals in the social instrumental task (SIT) and conditioned place preference (CPP). Behavioral experiments were conducted in a room with fixed low illumination (10–15 lux) and with controlled humidity (40%) and temperature (22°C–24°C). The experiments were always performed within a time frame that started approximately 2 h after the end of the dark circle and ended 2 h before the start of the next dark circle. All experiments were conducted under an authorization approved by Swiss law and the cantonal veterinary authorities.

### 2.2 Viruses

Viruses used here were previously described and validated (Bariselli et al. 2016; Bariselli et al. 2018; Contestabile et al. 2023; Tzanoulinou et al. 2022). Briefly, purified scr*Shank3* and sh*Shank3* (AAV1‐GFP‐U6‐scrmbshRNA; titre: 5.9 × 10^13^ GC/mL and AAV5‐ZacF‐U6‐luczsGreen‐sh*Shank3*; titer: 7.4 × 10^13^ GC/mL, Vector Biolabs) were injected in C57BL/6J mice. Viral injections in the NAc were delivered in mice at an early time point (at P5 or P6; < P6). The specificity and efficacy of the virus in the NAc were verified previously through tissue punches and qPCR analyses at P30 and through Western blotting after P60 (Tzanoulinou et al. 2022, see their sup. fig. 1). These analyses demonstrated that our shRNA approach leads to a significant and sustained reduction of *Shank3* mRNA and protein levels—specifically targeting the β‐ and γ‐isoforms—confined to the ventral striatum (i.e., NAc) and not affecting the dorsal striatum.

### 2.3 Stereotaxic Injections

**FIGURE 1:**
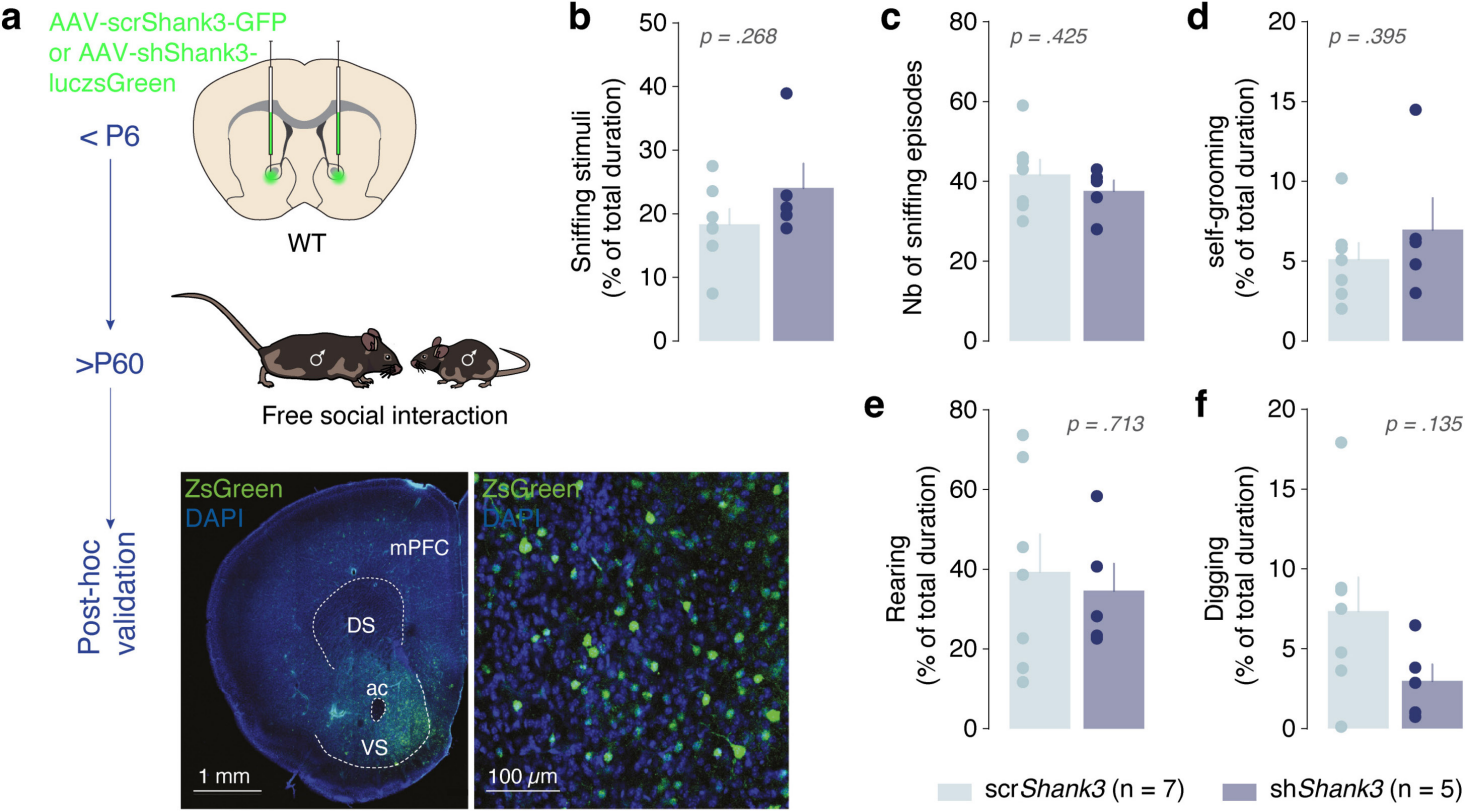
Postnatal downregulation of *Shank3* in the NAc does not impair free social interaction. (a) Schema and representative image of injection site in the NAc with AAV‐scrShank3‐GFP or AAV‐shShank3‐luczsGreen in P6 mice. Mice were then tested in a free social interaction test during adulthood. Bar graphs for scr‐ (light blue, *n* = 7) and sh*Shank3* (dark blue, *n* = 5) mice reporting the percentage of time. (b) Sniffing the sex‐matched juvenile conspecific (Mann–Whitney *U* test: *p* = 0.268). (c) Number of episodes sniffing the sex‐matched juvenile conspecific (unpaired *t*‐test: *t*_(10)_ = 0.832, *p* = 0.425). (d) Self‐grooming (unpaired *t*‐test: *t*_(10)_ = 0.909, *p* = 0.395), (e) rearing (unpaired *t*‐test: *t*_(10)_ = 0.378, *p* = 0.713), or (f) digging (unpaired *t*‐test: *t*_(10)_ = 1.626, *p* = 0.135) during the free social interaction test. All data are shown as the mean ± SE.

After anesthesia induction with a mixture of isoflurane/O_2_, C57Bl/6J wildtype pups were placed on a stereotaxic frame (Angle One; Leica, Germany). Mice were then locally anaesthetized with 50 μL lidocaine 0.5%, disinfected with betadine, and a small cut was performed. A quantity of 100 nL of scr‐ (scrambled—for control mice) or sh*Shank3* virus was injected bilaterally via a glass micropipette into the NAc (AP: +3.5 mm, ML: ±0.8 mm, DV: −3.2 mm; measured from lambda, as the bregma is not clearly visible at P5–P6). The surgical procedure was kept as brief as possible, lasting between 20 and 30 min, including anesthesia induction, viral injection, and wound closure. Pups were carefully monitored during recovery on a heating pad and were immediately returned to the dam afterwards. Maternal behavior post‐surgery was routinely monitored to exclude neglect or other abnormalities. Injection site validation was performed visually post hoc by two experimenters, confirming bilateral, region‐specific viral expression in the NAc by detecting fluorescent green markers carried by the viral vectors. A representative image meeting our inclusion criteria is shown in Figure 1. Further validation of injection sites was performed previously in separate cohorts as mentioned in Section 2.2 by Western blots and qPCR (Tzanoulinou et al. 2022).

### 2.4 Free Social Interaction

Experimental scr‐ and sh*Shank3* male mice were allowed to interact for 10 min with a sex‐matched juvenile mouse in a home cage environment with fresh bedding. Video recordings were scored offline and blindly with custom‐made software (Clicker, EPFL, Switzerland) and the behaviors denoted included social sniffing, rearing, digging, and self‐grooming.

### 2.5 Social Instrumental Task (SIT)

SIT was performed as previously described (Solie et al. 2022; Martin and Iceberg 2015). Briefly, the operant chamber (Med Associates) was composed of two different compartments divided by a gridded auto‐guillotine door: one chamber of 28 cm × 16 cm × 21 cm, where the experimental mice had ad libitum access to a lever press on the wall opposite to the door, and one chamber of 14 cm × 16 cm × 21 cm containing the social stimulus (unfamiliar sex‐matched younger mice, C57BL/6J). Upon one lever press, the gridded auto‐guillotine door opened for 7 s, allowing interaction between the experimental mouse and the social stimulus. The grid prevented passage of the animals between chambers. After 7 s, the door closed and the lever press could be repeated. During the whole session, the experimental animals had unlimited access to the lever, and thus, they could control the social interactions with the younger mouse. In line with previous SIT protocols (Solie et al. 2022; Martin and Iceberg 2015), the setup used here included one lever (active) per session. After each session, the apparatus was cleaned using 70% ethanol. The videos were recorded with an overhead camera at 25 fps and synchronized with the operant chamber TTL signal using the Neuralynx system (Digital Lynx 4SX acquisition system).

All the mice were isolated 1 week before the first session of the experiment to promote motivation to interact. The task was performed with one daily session of 20 min and divided into the following phases:

- *Shaping phase* (from day 1 to day 10): The animals were trained to associate the lever press with the opening of the door and, consequently, social interaction. Every time the animals were in proximity to the lever, the experimenter, through the Med Associates software, opened the door. Every day, the area around the lever was decreased, and on the last day of the shaping phase, the door was opened only when the mice were touching the lever.
- *Instrumental phase* (from day 11 to day 25): The animals had to perform the task by pressing the lever. One lever press opened the door to allow interaction with the conspecific for 7 s (fixed ratio 1).

“Learner” vs. “nonlearner” mice were classified as such based on their lever presses during the instrumental phase, with “learner” mice pressing the lever at least 10 times for three consecutive days (Solie et al. 2022; Martin and Iceberg 2015).

### 2.6 Social Conditioned Place Preference (CPP)

The protocol for social CPP was performed as previously described (Bariselli et al. 2018). The apparatus (Bioseb, Model BIOSEB, in vivo Research Instruments, spatial place preference box for mice LE895) used for the CPP protocol consists of two square‐shaped chambers (20 × 20 cm) with either grey stripes on a white background or black dots on a white background. The floor in each of the two chambers has different distinct textures. The two chambers are interconnected by a small corridor, and removable doors allow the corridor to be closed. After each session, the arena was cleaned thoroughly with 1% acetic acid. The protocol includes the following phases:

- *Day 0—Pre‐TEST*: experimental mice freely explored the CPP apparatus for 15 min to establish a baseline preference for both chambers. On the same day, stimulus mice were habituated for 15 min to the chamber used during conditioning sessions.
- *Days 1–4—Conditioning*: for each experimental mouse, one chamber was randomly assigned as the paired session chamber, with the presence of a nonfamiliar mouse (US+) and the other as the nonsocial session chamber (US–). This protocol remained stable for each mouse throughout the conditioning sessions. Experimental mice were subjected to six alternating conditioning sessions of 5 min each, resulting in 30 min of total conditioning time per day. During each conditioning session, the experimental subjects were allowed to freely interact with social stimuli. To counteract bias formation, each day the starting session was counterbalanced across mice and alternated for each mouse. After the end of each 5‐min trial, the animals were gently guided by the experimenter through the corridor and toward the other chamber; therefore, the animals were exposed to the spatial orientation of both chambers and apparatus.
- *Day 5—Post‐TEST*: similarly to the pre‐TEST, experimental mice were placed in the corridor of the CPP apparatus and, after lifting the removable doors, the animals could freely explore the arena once again for 15 min and establish a preference in the absence of the US.

The videos were recorded with an overhead camera at 25 fps using EthoVision XT 11 (Noldus, Wageningen, the Netherlands) for acquisition.

### 2.7 Animal Tracking

Bodypoints coordinates were tracked using DeepLabCut (DLC v2.3.3 (Mathis et al. 2018). The DLC models were trained on 320 manually labelled frames for CPP and 1300 frames for SIT, systematically sampled from 16 representative videos for CPP and SIT, respectively, with 10 anatomically defined markers positioned at key anatomical landmarks (snout, left ear, right ear, neck, gravity point, left hip, right hip, tail base, middle tail, and tail tip; Figure 3f) for CPP and five key landmarks (nose, body‐center, tail‐base, door‐center, and lever) for SIT. The resulting ResNet‐50‐based pose estimation model achieved mean tracking errors of 0.25 and 0.57 cm (1.42 and 3.27 pixels) for CPP and 0.184 cm and 0.181 cm (2.01 and 1.97 pixels) for training and test sets, respectively (Mathis and Mathis 2020).

### 2.8 Behavior Analysis in SIT

Behavioral events were identified using both video tracking and TTL signals recorded by the acquisition system. Lever presses were detected from TTL signals, and each press triggered a door opening programmed to last 7 s. Video tracking enabled precise analysis of the animal's position and orientation throughout the session. To standardize chamber dimensions across sessions, body point coordinates were normalized, with fixed reference positions for the lever and social zones.

Zone occupancy (action zone near the lever, interaction zone near the door) was determined based on the animal's location relative to predefined spatial coordinates during specific time windows (i.e., door open vs. door closed). Lever press behavior was quantified by counting the total number of presses per session, serving as a measure of task learning. Door opening duration was calculated as the cumulative time the door remained open, reflecting the total opportunity for social interaction. Note that the number of lever presses and door openings may slightly differ due to technical constraints: multiple rapid presses could trigger only one door opening, and presses during the 7‐s door‐open period were ignored (refractory period).

Social reward utilization was assessed by computing the proportion of time spent in the interaction zone while the door was open, normalized by the total time spent in that zone regardless of door state. This measure accounts for general spatial preference and more directly reflects the animal's willingness to engage in social interaction when given the opportunity.

### 2.9 Behavior Analysis in CPP

Behavior was analyzed based on body center tracking from EthoVision to measure distance moved and time spent in each chamber. Further behavioral analysis was performed with DeepLabCut tracking using VAME (Variational Animal Motion Embedding; (Luxem et al. 2022), trained on 120 videos with the following hyperparameters: test fraction = 0.1, temporal window = 30 frames, latent dimensions = 30. The model converged after 50 training epochs. Using VAME, we identified 41 distinct behavioral patterns that occurred in > 1% of time points across all sessions. Motifs were classified according to their latent dimensions and annotated by two behavioral experts.

### 2.10 Statistical Analyses

All behavioral scoring and data analyses were performed under blinded conditions. Specifically, the experimenters responsible for assessing behavioral outcomes were unaware of the group allocations throughout the data collection and scoring processes. Shapiro–Wilk analysis was used to assess the normality of sample distributions. *t*‐test with Welch's correction and paired *t*‐test were used for comparisons between two sample groups where appropriate and as indicated in the figure legends. When the normality was violated, nonparametric Mann–Whitney and Wilcoxon rank‐tests were applied. For multifactorial analysis, repeated‐measures two‐way ANOVA was performed, and *p* values of main effects and interaction are reported in the figure legends for each experiment. After significant main effects or interactions were revealed, post hoc tests were used for between/within group comparisons and reported in the figure legends or graphs. To determine whether motif usage in the social and empty conditions significantly deviated from equality, we performed a paired statistical analysis comparing the average motif usage between conditions. Motif usage data were aggregated to calculate the mean and standard error (SE) for each condition (social and empty) within defined experimental groups. The difference between the mean usage in the two conditions was computed for each motif. A paired *t*‐test was conducted to evaluate whether the observed differences significantly deviated from the diagonal. Significance level was set at *p* < 0.05. Data were analyzed using SPSS, GraphPad Prism, or R. Statistical analyses and values are reported in figure legends.

## 3 Results

### 3.1 Early Postnatal Downregulation of *Shank3* in the NAc Does Not Affect Social Exploration in a Free Interaction Assay

The NAc is a critical hub for reward processing and multiple other functions, integrating motivational and reward‐related signals to guide behavior (Floresco 2015; Castro and Bruchas 2019). Given its established vulnerability to *Shank3* perturbations and its role in social and nonsocial reward processing, we first investigated whether *Shank3‐NAcKD* impacts general social interaction. To achieve selective *Shank3* downregulation in the NAc, we employed an adeno‐associated viral‐mediated RNA interference approach, as previously described (Tzanoulinou et al. 2022). Experimental mice (sh*Shank3*) received AAV expressing short hairpin RNA targeting *Shank3*, while control mice (scr*Shank3*) were injected with a scrambled version of the virus (Figure 1a). Viral injections were performed between postnatal days 5 and 6 (P5–P6) to ensure downregulation occurred during a specific developmental window, shown previously to be particularly sensitive to perturbations linked to *Shank3* expression (Bariselli et al. 2016).

To evaluate the impact of *Shank3* downregulation on social interaction with a conspecific, in the absence of other salient stimuli, sh*Shank3* and scr*Shank3* mice were tested in a home cage environment during a 10‐min free social interaction paradigm with an unfamiliar sex‐matched juvenile mouse (Figure 1a). No significant differences were observed between groups in the set of behaviors annotated during social interaction, including sniffing the conspecific (Figure 1b,c). This suggests that early *Shank3‐NAcKD* does not significantly affect social interactions and possibly social reward processing in this setting. Additionally, no differences were found in other stereotypic or exploratory behaviors such as self‐grooming, rearing, or digging (Figure 1d–f).

### 3.2 Downregulation of *Shank3* in the NAc During Early Life Leads to Impairments in a SIT

**FIGURE 2:**
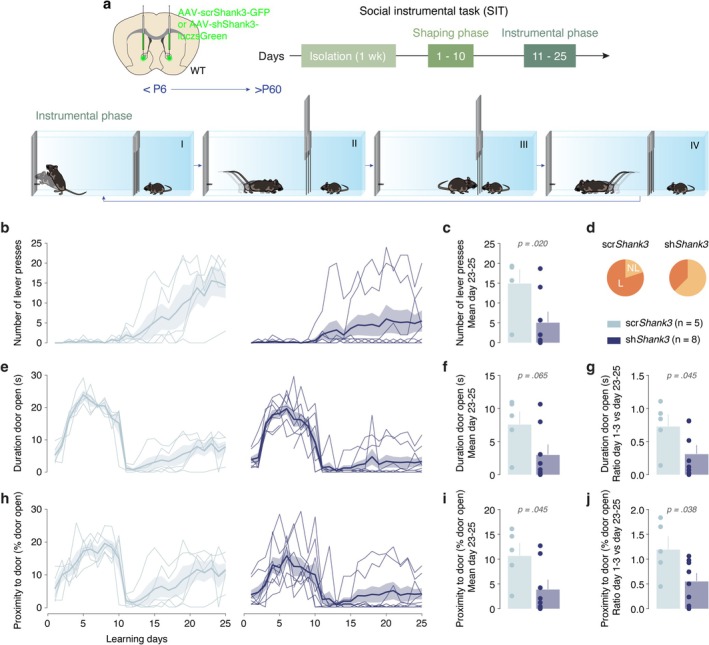
Deficits of Shank3 expression in the NAc result in impaired learning during a social instrumental task (SIT). (a) Top: schema of injection sites in the NAc with AAV‐scrShank3‐GFP or AAV‐shShank3‐luczsGreen in P6 mice. Mice were then tested in a social instrumental (SIT) task during adulthood. Bottom: schema of the SIT for one session. The mice can press the lever (I) to open a gridded auto‐guillotine door (II) and interact with a sex‐matched juvenile conspecific (III). The door stays open for 7 s before closing (IV). One session lasts 20 min, and the animals can press the lever at any time during the session. (b) Number of lever presses across days for scr‐ (light blue, *n* = 5) and sh*Shank3* (dark blue, *n* = 8). Unlike nonlearners (NL), learner (L) mice exhibit an increase in lever presses across sessions. (c) Bar graphs reporting the mean number of lever presses per scr‐ (light blue, *n* = 5) and sh*Shank3* (dark blue, *n* = 8) mice in the last 3 days of the task (day 23–25, Mann–Whitney U test: *p* = 0.020). (d) The proportions of L (learner, dark orange) and NL (non‐learners, light orange) mice are represented in pie charts. (e) Duration of door being open in seconds across days for scr‐ (light blue, *n* = 5) and sh*Shank3* (dark blue, *n* = 8). (f) Bar graphs reporting the mean duration of door in an open state for scr‐ and sh*Shank3* during the last 3 days of the task (day 23–25, Mann–Whitney U test: *p* = 0.065). (g) Bar graphs reporting the ratio of door open duration on the first three vs. the last 3 days of the task for scr‐ and sh*Shank3* (Mann–Whitney *U* test: *p* = 0.045). (h) Time spent near the door zone when open across days for scr‐ (light blue, *n* = 5) and sh*Shank3* (dark blue, *n* = 8). (i) Bar graphs reporting the mean duration of time spent near the door when open per scr‐ (light blue, *n* = 5) and sh*Shank3* (dark blue, *n* = 8) mice during the last 3 days of the task (day 23–25, Mann–Whitney *U* test: *p* = 0.045). (j) Bar graphs reporting the ratio of time spent near the door when open on the first three vs. the last 3 days of the task for scr‐ and sh*Shank3* (unpaired *t*‐test: *t*_(11)_ = 2.353, *p* = 0.038). Data are shown as the mean ± SE.

To assess the impact of *Shank3* downregulation in the NAc on social reward processing, we employed a well‐established SIT (Solie et al. 2022; Martin and Iceberg 2015). Experimental mice, injected early in postnatal life with scr‐ or sh‐ virus, were trained to associate lever pressing with door opening to access the social stimulus during daily 20‐min sessions that included a shaping and an instrumental phase (Figure 2a). The operant conditioning was considered successfully learned when the mice were pressing the lever at least 10 times for three consecutive days (Solie et al. 2022).

Notably, a clear distinction emerged when analyzing performance between groups. While most scr*Shank3* mice progressively increased the number of lever presses to gain access to social interaction as the training progressed, sh*Shank3* mice showed a marked deficit (Figure 2b). This difference in lever pressing between the groups remained evident even during the last 3 days of testing (day 23–25), with the majority of sh*Shank3* mice performing significantly fewer lever presses than controls during the instrumental phase, when lever pressing was required to obtain social interaction (Figure 2c). This was also reflected in the percentage of learners vs. nonlearners mice in each of the groups (Figure 2d). To understand better the response pattern between scr*Shank3* and sh*Shank3* with regard to reward opportunity, we examined the duration of the door being open (Figure 2e). During the shaping phase, experimenters opened the doors without lever pressing required from the animals and this is evident in the increased duration of door opening for both groups during days 1–10. Whereas there was a trend towards shorter duration of the door being open during the last 3 days of the SIT task for sh*Shank3* mice (Figure 2f), scr*Shank3* mice displayed a higher ratio for the door being open on the early shaping vs. late instrumental phase (days 1–3 vs. days 23–25) (Figure 2g). Finally, to assess the utilization of social reward when given the opportunity to interact with their younger conspecific, we quantified the time spent near the door when it was open (Figure 2h). When given opportunities, sh*Shank3* mice utilized significantly fewer rewards than scr*Shank3* mice during the last 3 days of training (Figure 2i). Moreover, whereas scr*Shank3* seemed to improve reward utilization over time, the ratio of early shaping vs. late instrumental phase is lower for the sh*Shank3* mice (Figure 2j).

This significant reduction in lever pressing observed in sh*Shank3* mice compared with controls could suggest deficits in the association between lever pressing and social reward availability, either due to social motivation‐related deficits revealed in the context of effort‐based behavior or due to a generalized deficit in instrumental conditioning. Moreover, given the reduced social reward utilization observed in sh*Shank3* mice, our results further support the interpretation of impaired motivation for social interactions after early NAc *Shank3* downregulation.

### 3.3 Impaired Social Reward‐Induced Learning in a CPP Assay

**FIGURE 3:**
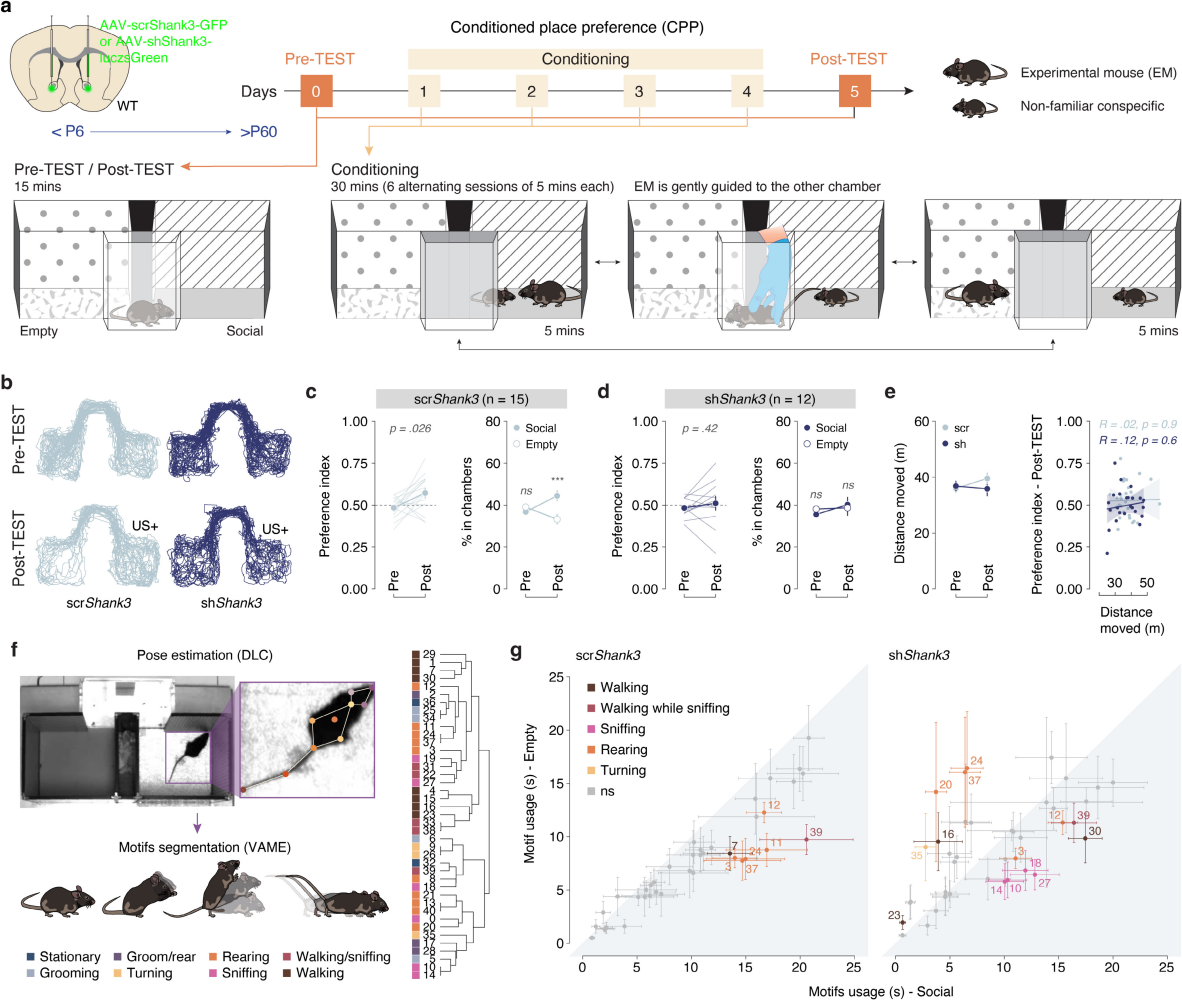
*Shank3* expression deficits in the NAc lead to impaired association in a conditioned place preference (CPP) task. (a) Top: schema of injection sites in the NAc with AAV‐scrShank3‐GFP or AAV‐shShank3‐luczsGreen in P6 mice. Mice were then tested in a conditioned place preference (CPP) task during adulthood. Bottom: schema illustrating the CPP procedure and apparatus. The protocol included a 15‐min pre‐test, a conditioning phase (30 min per day over four consecutive days), and a 15‐min post‐test (cf. Materials and Methods for more details). (b) Representative apparatus occupancy per scr‐ (light blue) and sh*Shank3* (dark blue) mice during pre‐TEST and post‐TEST. (c) Left: preference index calculated at pre‐ and post‐TEST for scr*Shank3* mice (*n* = 15, Wilcoxon signed‐rank test: *p* = 0.026). Right: percentage of time passed in the chambers for scr*Shank3* mice (*n* = 15, two‐way ANOVA: phase × time in chamber interaction: *F*_(1, 56)_ = 12.69, *p* = 0.0008; phase main effect: *F*_(1, 56)_ = 0.1531, *p* = 0.697; time in chamber main effect: *F*_(1, 56)_ = 4.977, *p* = 0.0297, followed by Bonferroni's multiple comparisons test). (d) Left: preference index calculated at pre‐ and post‐TEST for sh*Shank3* mice (*n* = 12, Wilcoxon signed‐rank test: *p* = 0.42). Right: percentage of time passed in the chambers for sh*Shank3* mice (*n* = 12, two‐way ANOVA: phase × time in chamber interaction: *F*_(1, 44)_ = 0.605, *p* = 0.441; phase main effect: *F*_(1, 44)_ = 0.983, *p* = 0.327; time in chamber main effect: *F*_(1, 44)_ = 0.038, *p* = 0.847, followed by Bonferroni's multiple comparisons test). (e) Left: distance moved measured during pre‐ and post‐TEST for scr‐ (light blue, *n* = 15) and sh*Shank3* (dark blue, *n* = 12) mice (two‐way ANOVA: phase × group interaction: *F*_(1, 46)_ = 0.1.786, *p* = 0.188; phase main effect: *F*_(1, 46)_ = 0.937, *p* = 0.338; group main effect: *F*_(1, 46)_ = 2.351, *p* = 0.1321, followed by Bonferroni's multiple comparisons test). Right: Correlations between distance moved and preference index in the post‐TEST for scr‐ (light blue, *n* = 15) and sh*Shank3* (dark blue, *n* = 12) mice. Pearson correlation coefficients and relative *p* values are reported per both groups. (f) Left: Schematic illustration of the video analysis. Pose estimation of 10 body parts with DeepLabCut and consecutive segmentation of the mice behavior in motifs with VAME. Right: Dendrogram reporting the clustering and behavioral annotations of the 41 identified motifs. (g) Motif usage in the empty versus social‐associated chambers is shown for scr‐ (left, *n* = 15) and shShank3 (right, *n* = 12) mice during the post‐TEST phase. Each point represents the mean motif usage per group, with the corresponding SEM. The difference between mean usage in the two conditions was calculated for each motif, and a paired *t*‐test was performed to assess whether the differences significantly deviated from the diagonal. Only motifs with significant differences in usage are highlighted, with colors corresponding to the behavioral annotations described in panel (f). All the data are shown as the mean ± SE.

The well‐established role of the ventral striatum in instrumental conditioning (Yin et al. 2008; Balleine et al. 2009), along with previous findings showing that *Shank3* homozygous mutant mice exhibit deficits in lever‐pressing tasks for food rewards (Wang et al. 2017; Jaramillo et al. 2016) prompted us to further examine social conditioning aspects after *Shank3‐NAcKD*. Specifically, we reasoned that the reduction in lever pressing observed in sh*Shank3* mice could be due to a broader instrumental performance deficit, or indeed impairments in social conditioning processes. To clarify this, we employed a social CPP assay (Figure 3a), a paradigm specifically designed to evaluate the reinforcing properties of social interaction with a nonfamiliar younger mouse (Bariselli et al. 2018). This approach relies on the innate rewarding properties of social interactions, using locomotion as the primary behavioral readout, thereby avoiding the confounds of instrumental task performance (Trezza et al. 2011; Dölen et al. 2013). Preference for the social stimulus‐paired chamber during the post‐test served as a measure of social reward‐based learning.

While scr*Shank3* mice spent significantly more time in the chamber associated with social stimuli during the post‐test, sh*Shank3* mice failed to develop this preference (Figure 3b–d). Importantly, no differences were observed between groups in the total distance moved, and no correlation was found between distance moved and the preference index (Figure 3e). These findings confirm that the impairment in sh*Shank3* mice is specific to the acquisition of the social CPP and not attributable to differences in locomotion or their initial motivation to explore the chambers. Together with the SIT results, these data suggest that the deficits observed in sh*Shank3* mice reflect impaired social reward conditioning. To gain deeper insights into potential behavioral differences during the CPP assay, we performed an analysis of locomotor motifs to assess exploratory strategies during the post‐TEST session in the absence of social stimuli. Using DeepLabCut (Mathis et al. 2018) for tracking 10 body points and VAME (Luxem et al. 2022) for behavioral classification (cf. Materials and Methods), we identified 41 discrete motifs exhibited by the mice (Figure 3f). Control mice showed a significantly increased usage of specific motifs (3, 7, 11, 12, 24, 37, and 39) within the chamber associated with the social stimulus (Figure 3g). These motifs appeared to correspond to exploratory behaviors, principally used to investigate the environment, such as walking, and walking while sniffing and rearing during the test phase. Interestingly, sh*Shank3* mice displayed a different pattern. While they preferentially employed certain “exploratory motifs” in the chamber paired with the social stimulus similarly to control mice (motifs 3, 12, and 39), sh*Shank3* mice also exhibited a significant preference for using some of these motifs in the unpaired (empty) chamber (motifs 24 and 37, Figure 3g right). Moreover, sh*Shank3* mice exhibited chamber‐specific preferences for additional motifs. More precisely, they showed increased use of walking‐related motifs (16 and 23) in the chamber associated with no stimulus and displayed a unique preference for sniffing‐related motifs (10, 14, 18, and 27) in the social‐associated chamber—a pattern absent in control mice. These differences suggest that early postnatal *Shank3‐NAcKD* alters how mice allocate exploratory behaviors between environments with different saliency. While controls showed focused investigation in the social‐paired chamber, shShank3 mice displayed more distributed exploration, indicating potential deficits in social associative conditioning.

These findings highlight the critical role of *Shank3* in the ventral striatum for both instrumental and associative social reward paradigms and provide new insights into how *Shank3* dysfunction impacts social behavior, shedding light on its contribution to the social deficits observed in neurodevelopmental disorders like ASD.

## 4 Discussion

Our results suggest that selective NAc *Shank3* downregulation in early postnatal life induces impairments in conditioning processes that involve social stimuli. Specifically, we show that sh*Shank3* mice did not lever press efficiently for having access to interactions with a juvenile mouse in a SIT. These findings were strengthened by our results in a CPP task, where sh*Shank3* mice showed impairments in forming conditioned‐to‐unconditioned stimuli (CS‐US) associations efficiently (i.e., associate the conditioned chamber with the presence of a rewarding social stimulus).

In both SIT and CPP tasks, the unconditioned stimuli were unfamiliar, sex‐matched juvenile mice, widely recognized as rewarding stimuli for mice, driving social‐seeking behavior. The rewarding properties of such a social stimulus were demonstrated not only behaviorally (Solie et al. 2022; Bariselli et al. 2018) but also neuronally, as firing of dopamine (DA) neurons is increased upon approach to the social target (Solie et al. 2022; Contestabile et al. 2021). Interestingly, previous validation experiments of our CPP procedure showed that it is not only the novelty component of the social stimulus that promotes conditioning but also the fact that the stimuli mice are juveniles and supposedly do not trigger antagonistic or aggressive behaviors (Bariselli et al. 2018). Moreover, Solié et al. demonstrated that activity of VTA DA neurons encodes social interaction and social‐induced reinforcement learning in the SIT, showing reward prediction error neuronal firing patterns (Solie et al. 2022). Thus, while novelty seems to play an important role in motivation for social interactions, it does not seem to be the only contributing factor to promote social seeking and conditioning.

We have previously shown that early NAc *Shank3* downregulation impairs social preference in the three‐chamber task (Tzanoulinou et al. 2022) where sh*Shank3* mice spent less time exploring the social stimulus enclosure and more time near the empty enclosure (i.e., object). Increased interest in inanimate objects is a characteristic of autistic phenotypes (Vivanti and Nuske 2017; Pierce et al. 2011). Here, in a free social interaction setting, we report that sh*Shank3* mice explore a novel juvenile mouse at equal time as their control counterparts.

This may be explained by several factors: (1) the absence of competing stimuli, leaving the juvenile mouse as the sole focal point of attention; (2) the unrestricted access to all sensory modalities, enhancing exploratory behavior; and (3) the possibility that the juvenile mouse initiates interactions, prompting reciprocal behavior. Future studies with more precise behavioral paradigms are needed to dissect these possibilities.

Nevertheless, while differences between sh*Shank3* and control mice are evident in the structured three‐chamber task, they appear occluded in the free social interaction test, likely due to fewer constraints and a lack of competing stimuli. This interpretation is in accordance with reduced social reward utilization by sh*Shank3* mice in the SIT task, where mice are given the opportunity to interact with younger conspecifics through a grid. Moreover, our findings align with a recent pre‐print report using conditional *Shank3* knockdown in the NAc, which demonstrated sociability deficits in the three‐chamber test but no significant differences in dyadic interactions (Folkes et al. 2025).

Perturbations of *Shank3* expression in the VTA during early postnatal life also led to social behavior deficits and specifically in disruptions to the maintenance of motivation for social interactions (Bariselli et al. 2016). Moreover, VTA *Shank3* downregulation also results in accelerated extinction in the social‐induced CPP (Bariselli et al. 2018). Here, we report that NAc *Shank3* downregulation resulted in impaired CPP acquisition, shown by no development of preference for the social‐paired chamber. Differently from our data, it is reported that conditional knockdown of *Shank3* in the NAc leads to a conditioned place aversion for the social stimulus in a similar CPP procedure (Folkes et al. 2025). This discrepancy can be explained by differences in the behavioral protocol, the exact NAc area affected, or other reasons. However, both our and others' data denote that *Shank3* downregulation in the NAc disrupts social reward‐induced CPP.

Our behavioral segmentation and analyses of locomotor motifs suggest that there are distinct exploratory patterns in the aftermath of early *Shank3‐NAcKD* that were expressed differently in the conditioned vs. the unconditioned chamber. Interestingly, data from humans with ASD have revealed different exploratory profiles in patients, and while these patterns are hypothesized to be significant for a complete phenotypic understanding of ASD, this field remains rather unexplored (Pisula and Pisula 2024). These data emphasize the need for refined behavioral paradigms that account for individual variability and can be employed to investigate specific aspects of exploratory and social behavior.

Taken together, previous and current data highlight the importance of optimal *Shank3* expression in the mesolimbic dopamine pathway for an array of social behaviors. Additionally, these results emphasize the need to dissect the differential contributions of region‐specific *Shank3* manipulations that underscore the heterogeneity of the observed phenotypes in our animal models that could be relevant for the human condition (Supekar et al. 2018). Future work will have to address other potential sources of ASD heterogeneity by investigating the consequences of cell‐specific and *Shank3* isoform‐specific manipulations (Delling and Boeckers 2021).

Although our data suggest that NAc sh*Shank3* mice show impairments in tasks involving social interactions and reward association, we cannot exclude the influence of other underlying cognitive or motivational factors. Even though we have previously shown that NAc sh*Shank3* mice displayed intact social recognition memory in a social novelty task (Tzanoulinou et al. 2022), the observed deficits may reflect broader disruptions in task engagement, sensitivity to experimental paradigms, or social motivation. Thus, one limitation of our study is that with the current data, we cannot exclude a general instrumental conditioning deficit. Future experiments will be needed to disentangle these potential contributions and evaluate additional aspects of cognition, exploratory behavior, and reward‐related components in this model.

According to our previous work, downregulating *Shank3* during this early developmental period (< P6) leads to specific changes in excitatory transmission in the VTA (Bariselli et al. 2016). Specifically, recordings from putative dopaminergic cells infected with the sh construct revealed an increased AMPA/NMDA ratio and an increased rectification index, highlighting the critical role of *Shank3* in the maturation of excitatory transmission of VTA dopamine neurons. When Shank3 was downregulated in the same area at a later timepoint (P20–P24) these changes in excitatory transmission were not observed, suggesting that the consequences of *Shank3* manipulations are time‐dependent (Bariselli et al. 2016). When *Shank3* was downregulated in the NAc during the first postnatal week, we observed an increase in the excitability of D1 (but not D2) receptor‐expressing medium spiny neurons (D1R‐MSNs) (Tzanoulinou et al. 2022). Although we do not know whether these D1R‐MSN changes would be similar if *Shank3* was downregulated in adulthood, we do know that later life downregulation (i.e., at P90) does not result in social preference deficits (Tzanoulinou et al. 2022). It would be pertinent for future work to study the link between social conditioning alterations reported here and synaptic alterations at the level of neurotransmitters and neuromodulators under a developmental perspective.

Given that injections for *Shank3* downregulation were performed at postnatal days 5–6, a period when the brain is still developing and anatomical boundaries are less distinct, one limitation of our study could be the potential for off‐target effects in neighboring brain regions. While all brains included in this study were visually inspected based on established anatomical criteria to verify the specificity of injection sites, and localization was further validated in other cohorts by tissue punches from both the NAc (target) and the dorsal striatal region (neighbouring control) demonstrating selective reduction in our region of interest (Tzanoulinou et al. 2022), some degree of spread to adjacent areas remains possible. Thus, despite these controls, future studies employing cell‐type‐specific or projection‐specific targeting strategies will be valuable for more precisely delineating the effects of *Shank3* downregulation and minimizing potential off‐target influences.

Finally, it is critical to adapt these experiments to include female mice. Despite the higher prevalence of ASD in boys, current diagnostic tools and experimental designs may overlook female‐specific phenotypes (Napolitano et al. 2022). In fact, recent studies challenge this bias, demonstrating that excluding females may overlook critical aspects of autism's neurobiology, as Shank3^Δ4–22^ and Cntnap^−/−^ female mice showed similar synaptic alterations as the corresponding male mutant mice (Tripathi et al. 2024). Incorporating sex as a biological variable and including both sexes in basic research is essential for improving the translational relevance of ASD studies (Shansky 2024).

Taken together, our data demonstrate that early postnatal *Shank3* downregulation in the NAc disrupts key aspects of social behavior. These findings have translational implications for refining behavioral paradigms and investigating the mesolimbic pathway as a hub for ASD‐related deficits. They also highlight the need for more sophisticated experimental designs that account for individual performance variability, ensuring more accurate and comprehensive behavioral assessments.

## Author Contributions

**Alessandro Contestabile:** conceptualization, data curation, formal analysis, investigation, methodology, project administration, software, supervision, visualization, writing – original draft, writing – review and editing. **Giulia Casarotto:** conceptualization, data curation, formal analysis, investigation, methodology, project administration, software, supervision, writing – review and editing. **Benoit Girard:** conceptualization, data curation, formal analysis, investigation, methodology, project administration, software, supervision, writing – review and editing. **Beatrice Righetti:** data curation, investigation, writing – review and editing. **Clément Solié:** methodology, supervision, writing – review and editing. **Camilla Bellone:** conceptualization, funding acquisition, methodology, project administration, resources, supervision, writing – review and editing. **Stamatina Tzanoulinou:** conceptualization, data curation, formal analysis, funding acquisition, investigation, methodology, project administration, resources, supervision, writing – original draft, writing – review and editing.

## Conflicts of Interest

The authors declare no conflicts of interest.

## Peer Review

The peer review history for this article is available at https://www.webofscience.com/api/gateway/wos/peer‐review/10.1111/ejn.70203.

## Supporting information


**Table S1.** Supporting Information.

## Data Availability

Data and sources will be available in the following repository account: https://github.com/TzanoulinouS/Tzanoulinou_lab, and upon contacting the corresponding author.
