# Supplementary material for: Early Postnatal Shank3 Downregulation in the Nucleus Accumbens Impairs Performance in Social Conditioning Paradigms in Male Mice
Source: Eur J Neurosci. 2025 Aug 4;62(3):e70203. doi: 10.1111/ejn.70203 (PMC12319878; doi:10.1111/ejn.70203)
Supplement: Supplementary file 1 — Table S1. Supporting Information. [file EJN-62-0-s001.pdf]

Supplementary Table 1

Cohort	Derived from # of litters	N of mice	Sex	Behavioral test	Comment	Figures
Cohort 1	4	7 scrShank3; 5 shShank3	Male	Free social interaction	All mice from cohort 1 performed the free social interaction test. A subset of this cohort performed the 3-chamber test as reported in Fig. 1 of Tzanoulinou et al. 2022.	1
Cohort 2	8	15 scrShank3; 12 shShank3	Male	Social Conditioned Place Preference (CPP) & Social Instrumental Task (SIT)	All mice from cohort 2 performed the CPP test (Figure 3). A subset of this cohort performed the SIT test, reported here on Figure 2 (5 scrShank3; 8 shShank3). A subset of Cohort 2 performed the O-maze as reported in Sup. Fig. 2o-p of Tzanoulinou et al. 2022.	2, 3
